# MRI-based 2.5D deep learning and radiomics effectively predicted microvascular invasion and Ki-67 expression in hepatocellular carcinoma

**DOI:** 10.1371/journal.pone.0336579

**Published:** 2025-11-14

**Authors:** Hongmei Yu, Depeng Kong, Xiaojun Mo, Ju Huang, Jie Wu, Yang Wang, Feizhou Du

**Affiliations:** 1 Department of Radiology, Chinese People’s Liberation the General Hospital of Western Theater Command, Chengdu, China; 2 Department of Medical Equipment, The Second Affiliated Hospital of Chengdu Medical College, Nuclear Industry 416 Hospital, Chengdu, China; University of Pennsylvania Perelman School of Medicine, UNITED STATES OF AMERICA

## Abstract

**Objective:**

To develop and validate an integrated 2.5D deep learning (DL) and Radiomics model using gadoxetic acid-enhanced MRI hepatobiliary phase (HBP) images combined with clinical features for preoperative prediction of microvascular invasion (MVI) and high Ki-67 expression (>20%) dual positivity in hepatocellular carcinoma (HCC).

**Methods:**

This retrospective study included 235 pathologically confirmed HCC patients categorized as MVI/Ki-67 double-positive (n = 129) or non-double-positive (n = 106). Clinical data (tumor diameter, AFP, GGT, differentiation grade, etc.) and HBP MRI images were collected. Tumor ROIs were segmented on HBP images. A 2.5D DL approach utilized axial, sagittal, and coronal planes of the largest tumor cross-section. LASSO regression selected key features from clinical, radiomic, and DL feature sets. Multivariate logistic regression identified independent predictors, and a nomogram was built. Model performance was evaluated via ROC curves, calibration plots, DCA, confusion matrices, and waterfall plots. Assessment of early recurrence within 2 years after HCC surgery was performed using alpha-fetoprotein (AFP) levels and imaging examinations.

**Results:**

Significant intergroup differences existed in tumor diameter, AFP, GGT, and differentiation grade (**P* *< 0.05). LASSO selected 38 key features (7 clinical, 23 DL, 8 radiomic). Multivariate analysis confirmed the derived clinical feature score, DL_Radscore, and radiomics Radscore as independent predictors of dual positivity. The integrated nomogram model (combining 2.5D DL, radiomics, and clinical features) achieved optimal prediction performance: AUROC, sensitivity, specificity, precision, accuracy, and F1-score values of 0.939, 0.793, 0.940, 0.942, 0.859, and 0.861, respectively.Calibration curves demonstrated good agreement, and DCA indicated clinical utility. Furthermore, postoperative follow-up confirmed that the MVI/Ki-67 dual-positive group exhibited a significantly higher early recurrence rate compared to the non-dual-positive group (*P* < 0.05).

**Conclusion:**

The integrated MRI 2.5D DL model synergizing radiomics and clinical features surpasses single-modality models for preoperative prediction of MVI/Ki-67 dual positivity in HCC. This tool shows strong potential for enhancing HCC risk stratification and guiding personalized treatment planning.

## Introduction

Hepatocellular Carcinoma (HCC) is the sixth most common cancer and one of the deadliest malignancies worldwide. Despite the availability of various interventions including surgical resection—the first-line treatment for early- and intermediate-stage HCC, the disease’s 5-year postoperative recurrence rate remains as high as 70% [1,2]. Notably, HCC tumors exhibit high spatial heterogeneity and a complex ecosystem comprising the Tumor Microenvironment (TME), which could promote tumor proliferation, angiogenesis, and immune evasion. This phenomenon could significantly influence tumor onset and progression, ultimately leading to diverse clinical outcomes and poor patient prognoses. Consequently, developing early diagnostic methods for HCC would be imperative for improved treatment selection and survival outcomes. Microvascular Invasion (MVI)—the presence of cancer cells within the microvessels of peritumoral and intratumoral tissues—is a pathological hallmark reflecting tumor aggressiveness and is presently considered a major risk factor for early recurrence and poor prognosis following HCC treatment [3]. On the other hand, Ki-67, a cell proliferation-associated nuclear antigen, exhibited expression levels that correlated strongly with tumor differentiation, invasive/metastatic potential, and prognosis, making the Ki-67 Labeling Index (Ki-67 LI) a potentially key biomarker for assessing HCC aggressiveness and both recurrence and metastasis risks. Specifically, a higher Ki-67 LI correlated with greater tumor invasiveness and was inversely associated with survival outcomes [4]. The prognostic assessment of HCC using dual biomarkers (e.g., MVI and Ki-67 expression) was reported to surpass that of any single biomarker [5–9]. Moreover, the synergistic impact of MVI positivity and high Ki-67 expression could significantly elevate the risk of early HCC recurrence. Presently, HCC MVI and Ki-67 could be mainly acquired via invasive biopsy or pathological examination after surgical resection, posing risks of trauma and untimeliness. In this regard, it is noteworthy that the preoperative non-invasive prediction of HCC MVI/Ki-67 dual biological markers could enhance clinicians’ understanding of patient conditions, better informing the selection of tailored treatment plans and further improving patient Survival Rates (SRs).

With rapid advancements in Artificial Intelligence (AI) technologies, medical imaging analysis is increasingly incorporating multiple pertinent Machine Learning (ML) methodologies, enabling the in-depth mining of conventional medical imaging data. Currently, these AI-powered approaches are being utilized to diagnose cancer phenotypes, characterize TMEs, and evaluate patient prognosis [10–13]. However, conventional radiomics primarily relies on manual Region-of-Interest (ROI) delineation using 2D cross-sectional slices, potentially leading to loss of information from adjacent slices and incomplete tumor characterization. As a core branch of ML, 2.5D Deep Learning (DL) employs multiplanar joint modeling, decomposing volumetric medical images into orthogonal 2D slice sequences (axial, sagittal, and coronal). It leverages Convolutional Neural Networks (CNNs) to extract cross-planar features while fusing spatial information across planes, thus improving small lesion detection rates while significantly reducing GPU memory requirements. When integrated with demographic and genomic data, this technique could provide a noninvasive and reproducible solution for clinical challenges in HCC, including issues related to diagnosis, staging, treatment planning, and prognosis assessment, potentially optimizing medical decision-making and advancing precision medicine [14]. Besides demonstrating fundamental lesion characteristics and vascular supply, Gadolinium-Ethoxybenzyl-Diethylenetriamine Pentaacetic Acid (Gd-EOB-DTPA)-enhanced Magnetic Resonance Imaging (MRI) could also reflect the hepatocyte functional status via Hepatobiliary Phase (HBP) imaging, offering distinct advantages in HCC diagnosis and treatment evaluation [15,16]. Nonetheless, contrary to previous research [17–20], which has focused primarily on predicting single biomarkers (either MVI or Ki-67 expression) using MRI radiomics or DL approaches, this approach suffers from limitations in model robustness and clinical interpretability. Moreover, only a few investigations have employed 2.5D DL in the simultaneous prediction of dual biomarkers (MVI and Ki-67). Herein, we integrated 2.5D DL and Radiomics analysis of Gd-EOB-DTPA-enhanced MRI with clinical features to develop a novel nomogram, which was assessed for its potential to predict the dual-positive MVI/Ki-67 status in HCC patients.

## Materials and methods

### Study population

This retrospective study involved HCC patients recruited from The General Hospital of Western Theater Command and the Nuclear Industry 416 Hospital between 01/01 2017 and 31/05 2023. The inclusion criteria were: (1) Patients with histopathological confirmation of HCC, as well as Ki-67 Labeling Index (LI) and MVI status information from Immunohistochemical (IHC) staining; and (2) Patients who completed Gd-EOB-DTPA-enhanced MRI within 2 weeks before surgery and received no anticancer treatments (including radiotherapy, chemotherapy, transarterial chemoembolization, or targeted therapy) before MRI examination. On the other hand, the exclusion criteria were: (1) Patients with poor-quality or incomplete MRI data; (2) Patients with missing preoperative laboratory results; (3) Patients with MVIs (portal vein, hepatic vein, or inferior vena cava tumor thrombus); and (4) Patients with recurrent HCC or extrahepatic metastases.

### Clinical data collection

**Fig 1:**
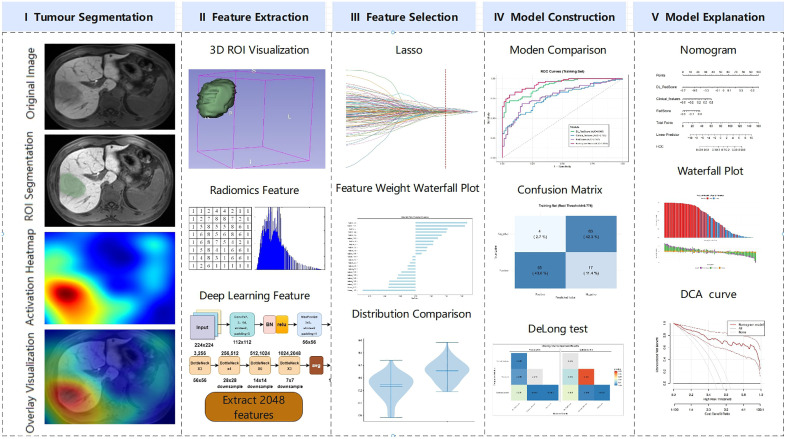
A workflow diagram depicting the integrated 2.5D DL, clinical, and radiomics approach for predicting the dual-positive MVI/Ki-67 status in HCC.

The following baseline data were collected from pathological records: (1) Demographic characteristics (age, sex, and hepatitis history); (2) Liver function parameters [Alanine Transaminase (ALT), Aspartate Transaminase (AST), Gamma-Glutamyl Transpeptidase (GGT), Albumin (ALB), and Total Bilirubin (TBIL)]; (3) Tumor markers [Alpha-Fetoprotein (AFP)]; (4) Hematological indices [Neutrophil Count (NC), Lymphocyte Count (LYC), Platelet Count (PLC), Neutrophil-to-Lymphocyte Ratio (NLR), and Platelet-to-Lymphocyte Ratio (PLR)]; (5)Tumor characteristics (maximum diameter, histological differentiation grade, MVI status, and Ki-67 LI). Based on a previously reported criterion [17], the patients were stratified into two groups by Ki-67 expression levels: Low-expression (Ki-67 ≤ 20%) and high-expression (Ki-67 > 20%). Furthermore, a dual-positive group was created for patients exhibiting both high Ki-67 expression and MVI positivity. On the other hand, non-dual-positive patients were further categorized into three subgroups: High Ki-67 expression + MVI-negative, low Ki-67 expression + MVI-positive, and low Ki-67 expression + MVI-negative. The Institutional Ethics Committee of our institution approved the study protocol (Approval No. 2022xjsxxm025), and the patients’ right to informed consent was waived due to the nature of the study. The data used in this study were accessed for research purposes on 15/05/2024. Fig 1 shows the study design and workflow.

### MRI acquisition protocol

Herein, MRI examinations were performed using a United Imaging uMR580 Zhiyun 1.5T scanner or a GE Architect 3.0T superconducting scanner, both equipped with a 16-channel abdominal phased-array coil. The standardized imaging protocol included: T1-Weighted Imaging (T1WI)-Repetition Time (TR) = 150 ms (1.5T)/155 ms (3.0T), Echo Time (TE) = 4 ms (1.5T)/5 ms (3.0T); T2-Weighted Imaging (T2WI)-TR = 1400 ms (1.5T)/1100 ms (3.0T), TE = 88 ms (1.5T)/74 ms (3.0T); and Fat-suppressed T1WI–TR = 4 ms (1.5T)/5 ms (3.0T), TE = 2 ms (both 1.5T and 3.0T scanners). All sequences were acquired with a slice thickness and interslice gap of 6 mm and 1 mm, respectively. After the intravenous administration of Gd-EOB-DTPA (Primovist, Bayer AG; 0.1 mL/kg), Dynamic Contrast-Enhanced (DCE) fat-suppressed T1WI was conducted in three phases: Arterial, portal venous, and equilibrium (TR = 4/5 ms, TE = 2/2 ms for all enhanced phases). Subsequently, HBP imaging (TR = 4 (1.5T)/5(3.0T) ms, TE = 2(1.5T)/2(3.0T) ms) was performed 30 min after contrast injection with a reduced slice thickness of 3 mm and an interslice gap of 1 mm.

### Image preprocessing, segmentation, and radiomic feature extraction

All MRI datasets [including B-spline interpolation for isotropic resampling (1 × 1 × 1 mm³ voxels), N4ITK-based bias field correction, z-score intensity normalization, and gray-level range standardization] in DICOM format were exported from the PACS system and preprocessed using 3D Slicer software. Two abdominal radiologists with over 5 years of experience (Reader A and Reader B) independently performed blinded volumetric ROI delineation on hepatobiliary phase images for the training cohort, carefully avoiding major vessels and bile ducts. Feature reproducibility was assessed using the Intraclass Correlation Coefficient (ICC), and only features with high inter-observer agreement (ICC > 0.8) were retained. For the external validation cohort, to avoid potential bias and simulate a realistic clinical workflow, all ROIs were delineated solely by Reader B, who was blinded to both clinical outcomes and training set segmentations.Using the PyRadiomics package (v3.0) in Python, we extracted 107 quantitative radiomic features from each ROI. Features with high inter-observer agreement (ICC > 0.8) were retained, including first-order statistics (n = 18) and 3D shape-based features (n = 14), Gray-Level Co-occurrence Matrix (GLCM, n = 24), Gray-Level Run-Length Matrix (GLRLM, n = 16), Gray-Level Size Zone Matrix (GLSZM, n = 16), Gray-Level Dependence Matrix (GLDM, n = 14), and Neighborhood Gray-Tone Difference Matrix (NGTDM, n = 5). To optimize feature selection, we employed an integrated approach involving Least Absolute Shrinkage and Selection Operator (LASSO) regression and Principal Component Analysis (PCA), ultimately obtaining a refined radiomics signature that was integrated into a comprehensive radiomics score (Radscore) via linear integration.

### DL feature extraction and selection

**Fig 2:**
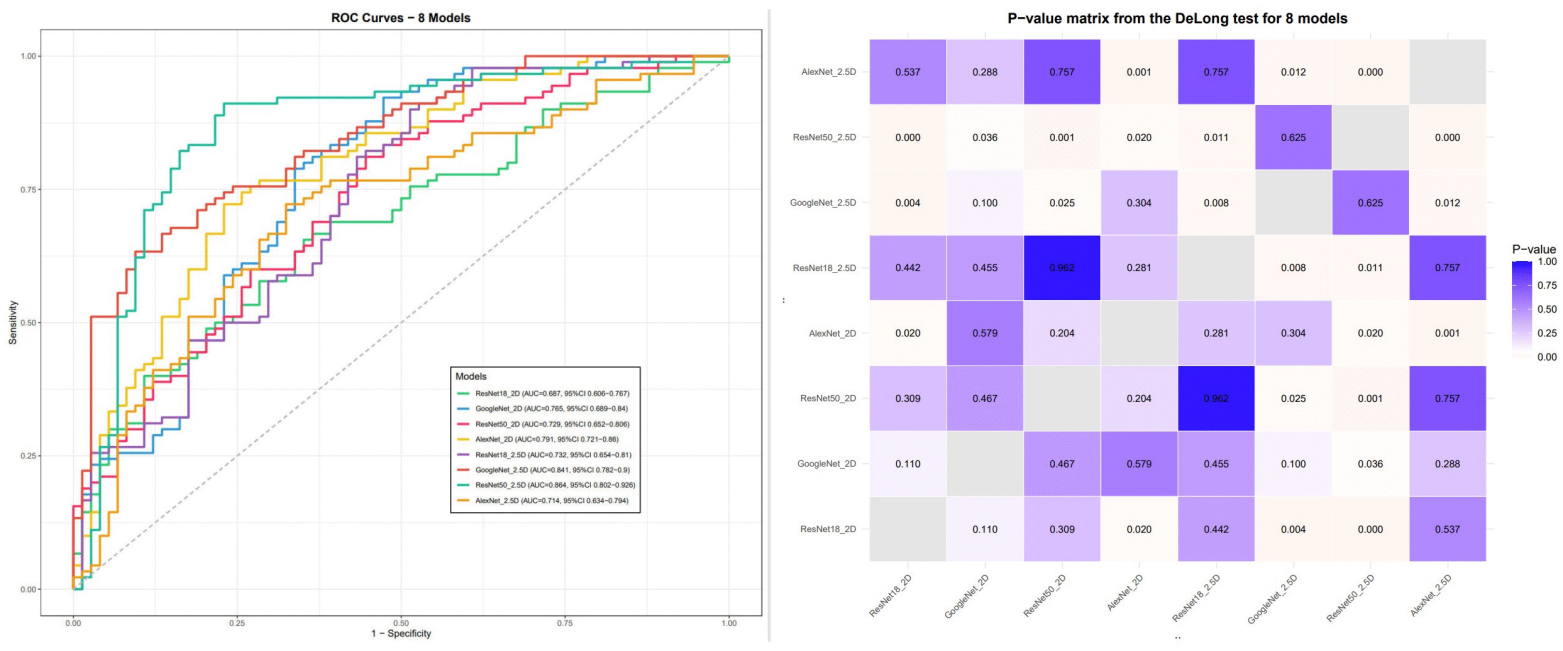
Screening of deep learning-based feature extraction models.

In MRI hepatobiliary phase analysis, deep learning features were developed through a dual approach where 2D features were extracted from the tumor’s largest cross-sectional slice, while adjacent multi-slice features along sagittal, coronal, and axial planes were assembled into 2.5D input blocks to capture spatial context, with each axial slice combined with its adjacent slices to form three-channel inputs that were normalized and resized to 224 × 224 pixels. Subsequently, self-supervised fine-tuning was applied to multiple pre-trained networks(AlexNet, GoogLeNet, ResNet18, and ResNet50) and comparison of ROC curves between 2D and 2.5D models using DeLong’s test demonstrated that ResNet50 achieved optimal performance under the 2.5D framework (training AUC: 0.864, 95% CI: 0.802–0.926), with 2.5D models significantly surpassing their 2D counterparts (Fig 2). Accordingly, ResNet50 was employed as the backbone architecture for 2.5D feature extraction, generating 2048-dimensional feature vectors per slice through truncation at the global average pooling layer. On the other hand, patient-level feature vectors were generated via average pooling across all slices. This approach, leveraging hierarchical feature aggregation and transfer learning, enabled dimensionality reduction from 3D medical images to 2D deep feature representations. For feature selection, Shapiro-Wilk and Leven’s tests were employed to assess normality and homogeneity of variance, with significant features (*P* < 0.05) identified using independent t-tests (normally distributed) or Wilcoxon rank-sum tests (non-normally distributed). We employed 5-fold cross-validation within the training set to tune the hyperparameter (λ) in the LASSO regression. This internal validation process helps to optimize the model’s complexity and reduce the risk of overfitting to the training data. Non-zero coefficient features were retained, and their performance was validated through K-Nearest Neighbors (KNN) cross-validation, yielding feature weights and a composite DL radiomics score (DL_Radscore).

### Construction of predictive signature scores

Following feature selection via LASSO regression, three distinct signature scores were constructed for each patient to integrate the selected features from each modality. Each score was calculated as a linear combination of the selected standardized features, weighted by their respective coefficients derived from the models.

Clinical Feature Score: The selected clinical variables were incorporated into a multivariate logistic regression model. Each variable was weighted by its corresponding regression coefficient (*β*). The Clinical Feature Score was computed as follows: Clinical Feature Score = *β*_*0 *_*+ β*_*1 *_**⋅* *C**_*1 *_*+ β*_*2*_* *⋅* *C**_*2*_* + *⋯* + β*_*p*_* *⋅* *C**_*p*_.

*Note: C*_*1*_*,C*_2_*,…,C*_*p*_ *represent the Z-score standardized continuous clinical variables or appropriately encoded categorical variables, and β*_*0*_*,β*_*1*_*,…,β*_*p*_ *denote the intercept and regression coefficients obtained from the multivariate logistic model.*

RadScore: The radiomics features with non-zero coefficients selected by LASSO regression were used to build the radiomics signature. The RadScore was defined as: RadScore = *α*_*0 *_*+ α*_*1 *_**⋅* *R**_*1 *_*+ α*_*2 *_**⋅* *R**_*2*_* + *⋯* + α*_*n *_**⋅* *R**_*n*_*.*

*Note: R*_*1*_*,R*_*2*_*,…,R*_*n*_ *are the Z-score standardized values of the selected radiomic features, and α*_*0*_*,α*_*1*_*,…,α*_*n*_ *are the corresponding non-zero coefficients from the LASSO model*.

DL_RadScore: Similarly, the selected deep learning features were used to construct this score. The formula applied was: DL_RadScore = *γ*_*0*_* + γ*_*1 *_**⋅* *D**_*1 *_*+ γ*_*2*_* *⋅* *D**_*2*_* + *⋯* + γ*_*m*_* *⋅* *D**_*m*_.

*Note: D*_*1*_*,D*_*2*_*,…,Dm represent the Z-score standardized deep learning features, and γ*_*0*_*,γ*_*1*_*,…,γ*_*m*_ *are the non-zero coefficients derived from the LASSO regression.*

These three scores—Clinical Feature Score, RadScore, and DL_RadScore—were subsequently used as independent variables in the development of the combined nomogram prediction model.

### Follow-up and recurrence assessment

Postoperative follow-up was conducted at 1- to 3-month intervals using laboratory tests (AFP) combined with imaging examinations (ultrasound, CT, or MRI) to assess tumor recurrence. Recurrence was defined by the presence of typical imaging features of HCC or reconfirmation via pathology. The follow-up period ended at 24 months postoperatively.

### Statistical analysis

Statistical analyses were performed using Python (version 3.9), R-4.3.3 (specifically the ggplot2, reshape, car, regplot, rms, pROC, and rmda packages), and SPSS 26.0. Normally distributed continuous variables were expressed as mean ± Standard Deviation (SD) (x ± s) and compared using t-tests, while non-normally distributed variables were presented as median (P25, P75) and analyzed with Mann-Whitney U tests. On the other hand, categorical variables were compared using the chi-square test. The most valuable clinical and radiomics features for predicting dual-positive MVI/Ki-67 status in HCC were identified through LASSO regression. Subsequently, significant variables from clinical, radiomics, and DL analyses were incorporated into multivariate logistic regression to construct a nomogram prediction model. The following approaches were used to assess model performance: 1) Waterfall plots, which visualized individual patient feature contributions and risk stratifications; 2) Confusion matrix metrics (sensitivity, specificity, accuracy, precision, and F1-score); 3) Receiver Operating Characteristic (ROC) curve analysis; 4) Calibration curves, which assessed prediction accuracy; and 5) Decision Curve Analysis (DCA), which evaluated clinical utility.

## Results

### Comparison of patients’ baseline characteristics and clinical features

Of the 291 HCC patients initially enrolled based on the inclusion criteria, 29, 10, 8, and 9 were excluded for poor MRI data quality/incomplete data, missing preoperative laboratory values, postoperative HCC recurrence, and HCC with tumor thrombus/extrahepatic metastasis, respectively. Consequently, only 235 HCC patients [including 149 from our institution as the training set (82 and 67 double-positive and non-double-positive cases, respectively) and 86 from the Nuclear Industry 416 Hospital as the external validation set (47 and 39 double-positive and non-double-positive cases, respectively) were included in the final analysis. The training and validation sets exhibited significant differences in serum parameters (NLR and TBIL) (*P* < 0.05), with no statistically significant differences observed in other baseline clinical characteristics or serum parameters (*P* > 0.05). Furthermore, the double-positive and non-double-positive groups revealed significant differences in tumor diameter, AFP, GGT, and differentiation grade (*P* < 0.05), with no significant differences observed in gender, age, hepatitis status, AST, ALT, NC, LYC, PLC, NLR, or PLR (*P* > 0.05) (Tables 1 and 2).

**Table 1:** Comparative analysis of the clinical characteristics of patients in the two groups [n, %]/[IQR], P25-P75.

Characteristic	Total (n = 235)	Training set (n = 149)	Validation set (n = 86)	Statistical value	*P-*value
**Sex**				0.096	0.756
**Male**	199 (84.7)	127 (85.2)	72 (83.7)		
**Female**	36 (15.3)	22 (14.8)	14 (16.3)		
**Age, years**	56.4 ± 9.8	55.6 ± 9.3	57.9 ± 10.4	1.788	0.075
**Hepatitis**				2.678	0.102
**Yes**	188 (80.0)	127 (85.2)	66 (76.7)		
**No**	47 (20.0)	22 (14.8)	20 (23.3)		
**Tumor size (cm)**	3.7 (2.2,6.5)	3.5 (2.0,6.5)	4.2(2.7,6.5)	−1.241	0.214
**AFP (ng/ml)**				0.081	0.960
**<20**	114 (48.5)	72 (48.3)	42 (48.8)		
**20 ~ 400**	64 (27.2)	40 (26.8)	24 (27.9)		
**>400**	57 (24.3)	37 (24.8)	20 (23.3)		
**Neutrophil**	3.22 (2.3,4.3)	3.4(2.4,4.3)	3.1 (2.3,4.2)	−0.603	0.547
**Lymphocyte**	1.3 (0.9,1.7)	1.3(0.9,1.6)	1.3 (1.0,1.9)	−1.892	0.059
**Platelet**	132 (102,185)	127(96, 188)	142 (107,185)	−1.354	0.176
**NLR**	2.5 (1.8,3.5)	2.7(2.0,3.6)	2.3 (1.7,3.0)	−2.210	0.027
**PLR**	102.7 (80,142.1)	105.2(79.0,149.1)	100.3 (79.8,134.5)	−1.067	0.286
**ALT**	37.5 (24.4,63.5)	40.8(24.5,64.1)	34.3 (24.2,61.2)	−0.946	0.344
**AST**	35.1 (27.2,59)	35.1(27.3,61.6)	35.0 (26.7,54.4)	−0.496	0.620
**GGT**	57.3 (31.9,110.6)	59.1(33.7,113.5)	56.3(29.2,106.7)	−0.664	0.506
**ALB**	43.1 (40.4,45.8)	43.1 (40.8,46.6)	42.8(40.1,44.6)	−1.141	0.254
**TBIL**	17.8 (13.2,24.3)	18.5(14.0,27.1)	16.4(12.8,21.8)	−2.109	0.035
**Differentiated degree**				0.139	0.933
**Poorly differentiated**	39 (16.6)	25 (16.8)	14(16.3)		
**Moderately differentiated**	158 (67.2)	99 (66.4)	59(68.6)		
**Highly differentiated**	38 (16.2)	25 (16.8)	13(15.1)		

**Table 2:** Comparative analysis of the clinical characteristics of patients in the two groups [n,%]/[IQR], P25–P75.

Characteristic	Training set(n = 149)				Validation set(n = 86)			
	Double positive group	Non-double positive group	Statistical value	*P* value	Double positive group	Non-double positive group	Statistical value	*P* value
**Sex**			0.002	0.960			0.626	0.429
**Male**	70(85.4)	57(85.1)			38(80.9)	34(87.2)		
**Female**	12(14.6)	10(14.9)			9(19.1)	5(12.8)		
**Age, years**	54.2 ± 10.0	57.1 ± 8.2	1.929	0.056	56.8 ± 9.3	59.2 ± 11.7	1.057	0.294
**Hepatitis**			0.264	0.607			0.227	0.633
**Yes**	71(86.6)	56(83.6)			37(78.7)	29(74.4)		
**No**	11(13.4)	11(16.4)			10(21.3)	10(25.6)		
**Tumor size (cm)**	4.2(2.6,8.1)	3.0(1.7,4.3)	−3.871	**<0.001**	4.7(3.4,7.0)	3.7(2.0,5.2)	−2.117	0.034
**AFP (ng/ml)**			17.661	**<0.001**			23.252	**<0.001**
**<20**	30(36.6)	42(62.7)			13(27.7)	29(74.3)		
**20 ~ 400**	21(25.6)	19(28.4)			15(31.9)	9(23.1)		
**>400**	31(37.8)	6(9.0)			19(40.4)	1(2.6)		
**Neutrophil**	3.3(2.5,4.3)	3.5(2.3,4.4)	−0.494	0.621	3.2(2.3,4.3)	3.0(2.3,4.1)	−0.2	0.842
**Lymphocyte**	1.2(0.9,1.6)	1.3(0.9,1.6)	−0.166	0.868	1.3(0.9,1.9)	1.3(1.1,1.9)	−0.633	0.527
**Platelet**	135(101,191)	114(92,183)	−1.361	0.174	131(103,167)	156(110,206)	−1.844	0.065
**NLR**	2.7(2.0,3.6)	2.7(2.0,3.6)	−0.052	0.959	2.3(1.9,2.9)	2.2(1.5,3.7)	−0.807	0.420
**PLR**	109.1(85.6,163.9)	102.0(70.0,138.8)	−1.360	0.174	96.3(80.3,121.8)	110.8(78.0,146.8)	−0.863	0.388
**ALT**	41.7(25.8,62.4)	40.8(22.0,65.8)	−0.141	0.888	34.2(25.1,55.4)	34.4(22.9,63.7)	−0.117	0.907
**AST**	34.6(27.8,61.2)	38.1(27.1,77.3)	−0.244	0.807	33.6(28.2,48.6)	38.6(24.5,58.2)	−0.399	0.690
**GGT**	77.1(42.6,126.8)	48.1(30.5,92.5)	−3.196	0.010	71.2(31.,170.1)	50.6(25.0,80.7)	−2.052	0.040
**ALB**	43.3(41.3,46.7)	42.6(39.5,46.6)	−1.473	0.141	42.1(39.7,44.7)	43.6(40.6,44.6)	−0.980	0.327
**TBIL**	18.0(13.1,24.9)	18.7(15.7,27.6)	−0.885	0.376	16.2(12.6,23.3)	16.5(13.0,21.6)	−0.056	0.955
**Differentiated degree**			14.429	0.001			10.197	0.006
**Poorly differentiated**	19(23.2)	6(9.0)			10(21.3)	4(10.3)		
**Moderately differentiated**	57(69.5)	42(62.7)			35(74.5)	24(61.5)		
**Highly differentiated**	6(7.3)	19(28.4)			2(4.3)	11(28.2)		

### Screening of clinical, DL, and radiomics features

**Fig 3:**
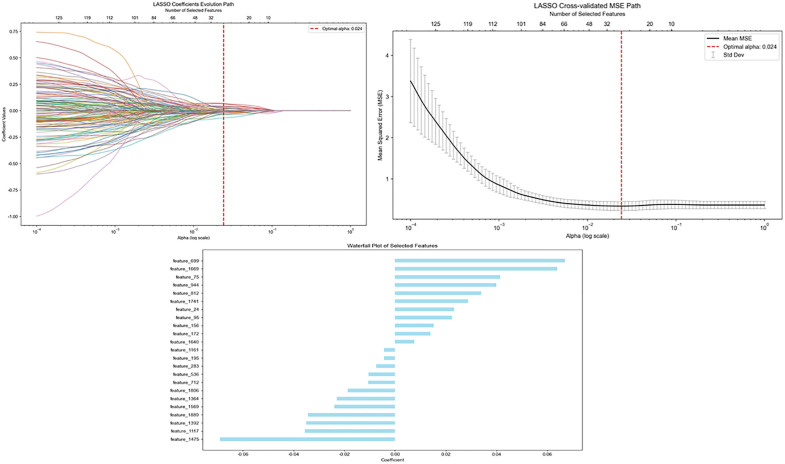
Displays the feature names and weights of the DL characteristics identified through the LASSO regression-based feature selection.

The delineation of MRI HBP images from all 235 HCC patients yielded 2048-dimensional DL features and 107 radiomics features, all of which were standardized using Z-score normalization. Highly collinear features were eliminated using Variance Inflation Factor (VIF < 5) screening. Subsequently, LASSO regression with the L1 regularization penalty (coefficient λ) was employed to identify clinical, DL, and radiomics features that correlated significantly with HCC MVI/Ki-67 dual positivity. Meanwhile, irrelevant features were compressed to zero coefficients via 5-fold cross-validated optimization. Finally, 38 key features, including 7 clinical features (Age, Diameter, AFP, ALB, AST, GGT, and differentiation degree), 8 radiomics features with non-zero coefficients (firstorder_Median, glrlm_LowGrayLevelRunEmphasis, gldm_DependenceEntropy, shape_Flatness, glszm_LargeAreaHighGrayLevelEmphasis, firstorder_10Percentile, shape_Maximum2DDiameterColumn, and glszm_LowGrayLevelZoneEmphasis), and 23 DL features, were selected. Fig 3 show the feature selection processes and corresponding weights, from which three composite scores were derived: Clinical features score, Radscore, and DL_Radscore.

### Development and performance evaluation of the nomogram prediction model

**Fig 4:**
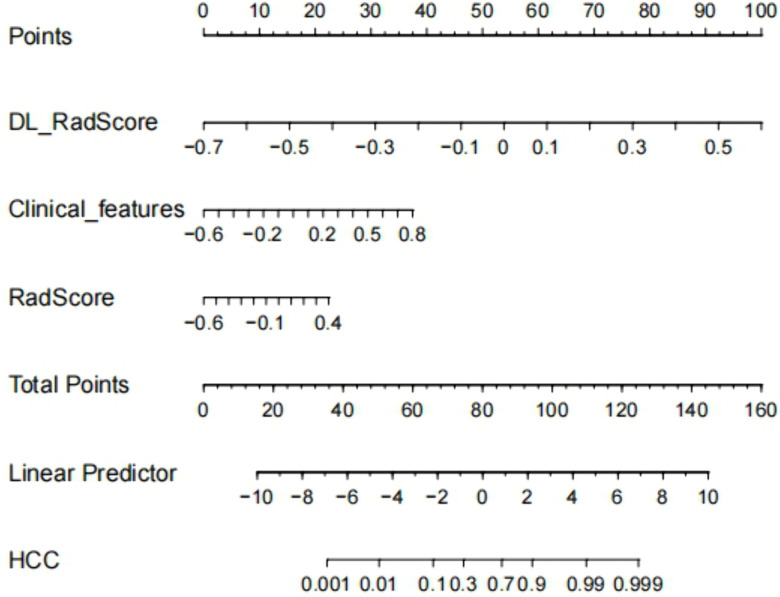
The nomogram integrating DL, radiomics, and clinical features for predicting MVI/Ki-67 dual positivity in HCC.

**Fig 5:**
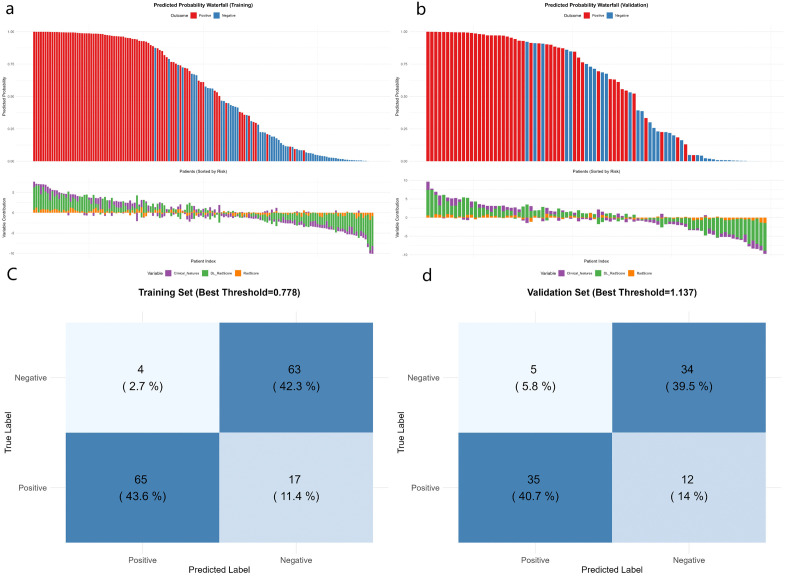
Predictive performance for HCC MVI/Ki-67 dual positivity: (a, b) Waterfall plot of individual models, and (c, d) Confusion matrix of the combined model.

**Fig 6:**
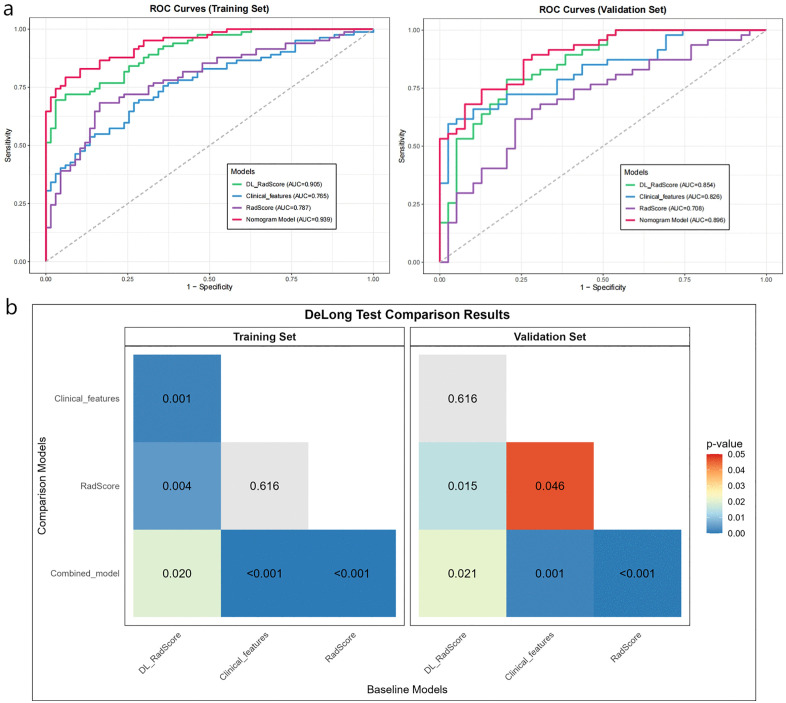
Comparison of predictive models for HCC MVI/Ki-67 dual positivity: (a, b) ROC curves of different models, and (c, d) DeLong’s test for statistical comparison of their diagnostic efficacy.

**Fig 7:**
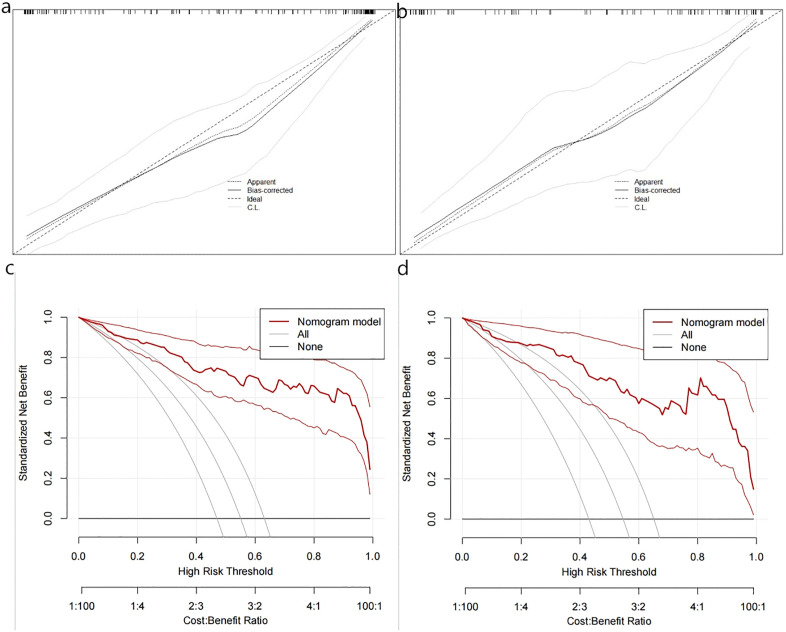
Evaluation of the combined model for predicting HCC MVI/Ki-67 dual positivity: (a, b) Calibration curve assessing prediction accuracy, and (c, d) Decision curve analysis (DCA) evaluating clinical utility.

Multivariate logistic regression analysis confirmed the Clinical features, DL_RadScore, and RadScore to be independent risk factors for HCC MVI/Ki-67 dual positivity. These parameters were used to construct the nomogram prediction model, in which the sum of individual component scores corresponded to the predicted probability of dual positivity (Fig 4). The waterfall plot effectively demonstrated model performance and individual feature contributions by strategically visualizing patients ranked by composite score (descending order). Color gradients and transparency reflected prediction confidence, while higher scores indicated greater confidence in dual-positive classification. This approach enabled the rapid identification of high-risk (leftmost) versus low-risk (rightmost) subgroups (Fig 5a, b). On the other hand, the confusion matrix confirmed reliable positive prediction with optimal balance between reduced misdiagnosis and missed diagnosis rates, thus confirming our model as an effective screening tool for dual-positive HCC (Fig 5c, d, Table 3). Notably, the clinical model performed slightly better in the external validation set (AUC = 0.826) than in the training set (AUC = 0.765). This could be attributed to the distinct distribution of clinical features (e.g., NLR, TBIL) between the two cohorts (Table 1), suggesting that the clinical model generalized well to the external population despite these difference.According to ROC analysis, the combined model (AUC: 0.939/0.896 in training/validation sets) demonstrated superior performance compared to individual DL (0.905/0.854), radiomics (0.878/0.708), or clinical feature (0.765/0.826) models in predicting both Ki-67 expression and MVI status (Fig 6a, b). DeLong’s test revealed that the combined model significantly outperformed both the clinical-only model and the radiomics-only model in both the training and external validation cohorts (all *P* < 0.05).(Fig 6c, d). Calibration curves demonstrated excellent agreement between predicted and observed probabilities, while DCA confirmed the model’s clinical utility across intermediate risk thresholds (1:4–4:1), supporting personalized therapeutic decision-making for HCC patients (Fig 7).

**Table 3:** Efficacy Analysis of the Clinical, DL_RadScore, Radiomics, and Combined Models in Diagnosing Double-Positive Ki-67 and MVI in HCC.

Models	Sets	AUC (95 CI)	Sensitivity	Specificity	Precision	Accuracy	F1-score
**Clinical features**	Training	0.765(0.690-0.840)	0.683	0.731	0.757	0.705	0.718
	Validation	0.826(0.740-0.912)	0.596	0.974	0.966	0.767	0.737
**DL_RadScore**	Training	0.905(0.861-0.950)	0.695	0.97	0.966	0.819	0.809
	Validation	0.854(0.775-0.934)	0.787	0.795	0.822	0.791	0.804
**RadScore**	Training	0.787(0.714-0.860)	0.683	0.836	0.836	0.752	0.752
	Validation	0.708(0.597-0.818)	0.617	0.769	0.763	0.686	0.682
**Combined Models**	Training	0.939(0.905-0.973)	0.793	0.94	0.942	0.859	0.861
	Validation	0.896(0.833-0.958)	0.745	0.872	0.875	0.802	0.805

### Prognostic assessment in HCC patients

In the training cohort, among 82 patients in the MVI/Ki-67 double-positive group, 5 had missing follow-up data, and 31 (40.3%) experienced early recurrence. Of the 67 patients in the non-double-positive group, 4 were lost to follow-up, and 12 (19.0%) showed early recurrence. In the external validation cohort, 18 of 47 (38.3%) patients in the double-positive group had early recurrence, while in the non-double-positive group (n = 39), 2 cases had missing data, and 6 (16.2%) exhibited early recurrence. The early recurrence rate was significantly higher in the double-positive group than in the non-double-positive group in both the training and validation sets, with a statistically significant difference (*P* < 0.05).

## Discussion

This study elucidates HCC as one of the most prevalent and rapidly progressing malignancies, with MVI/Ki-67 dual biomarkers serving as critical determinants of its prognosis and recurrence. Therefore, early detection of these biomarkers could better facilitate recurrence risk stratification, optimizing therapeutic decision-making and ultimately improving patients’ survival outcomes. Despite their usefulness in HCC diagnosis and prognosis assessment, conventional DL algorithms have demonstrated several limitations such as incomplete tumor information [21–23]. Consequently, we employed a 2.5D DL approach that extracts features across multiple planes (axial, sagittal, and coronal), not only compensating for the aforementioned limitation in single-view radiomics but also mitigating the substantial GPU memory demands of full 3D processing, thereby significantly enhancing the model’s clinical applicability. Herein, 235 HCC patients were included in the final analysis, which identified clinical features, DL signatures, and radiomics scores as significant predictors of dual positivity, based on which a nomogram prediction model was developed. Furthermore, waterfall plots were generated to visualize feature contributions, enhancing model interpretability and providing a reference for individualized risk assessment. Furthermore, our multimodal framework—integrating MRI-based 2.5D DL, as well as radiomics and clinical features—may noninvasively predict HCC MVI/Ki-67 dual biomarkers preoperatively, thus overcoming limitations of invasive biopsies or postoperative pathological examinations. It could also facilitate personalized treatment planning and more precise prognostic evaluation.

Some characteristic features of HCC include Tumor Microenvironment (TME) alterations such as intratumoral necrosis, chronic inflammation, and cellular apoptosis, which could lead to immune cell infiltration and inflammatory cytokine release. Notably, inflammatory indices could accelerate tumor growth, as well as promote angiogenesis and MVI progression, thus highlighting their correlation with HCC prognosis [24,25]. Herein, demographic information, as well as tumor (diameter, differentiation grade), inflammatory, and hepatic function parameters were examined, revealing statistically significant intergroup differences in tumor diameter, AFP, GGT, and differentiation grade (*P* < 0.05). Furthermore, consistent with previous research [17,26–28], the LASSO-selected features and multivariate logistic regression confirmed tumor diameter, AFP, GGT, and the differentiation grade as significant predictors of HCC MVI/Ki-67 dual positivity. Additionally, AFP, an established biomarker for HCC screening, diagnosis, and surveillance, demonstrated some predictive value for dual positivity, with patients showing a significantly more dual-positive status also exhibiting serum AFP levels > 400 ng/ml.Other established markers such as Des-gamma-carboxy prothrombin (DCP) and AFP-L3 fraction have shown promising value in HCC diagnosis and prognosis prediction [29]. Incorporating these biomarkers in future studies could potentially enhance the predictive power of the clinical component of our model. We also established that liver function markers (ALT, AST, GGT, ALB, and TBIL) could reflect hepatocyte integrity, biliary excretion, and synthetic function, potentially influencing tumor invasiveness and prognosis. This phenomenon implies that the aforementioned markers could elucidate pathophysiological differences among hepatitis, cirrhosis, and HCC patients [30]. Moreover, tumor diameter correlated directly with aggressive biological behavior, with heightened invasiveness and proliferative capacity potentially driving rapid growth. Histological differentiation, a critical pathological indicator of malignancy, also directly influenced biological aggressiveness (including invasion and metastatic potential). This finding particularly aligns with previous research which linked marked cellular atypia, stronger invasiveness, higher MVI/Ki-67 co-expression risk, and increased postoperative recurrence/metastasis with poorly differentiated HCC [31]. Notably, no other laboratory parameters showed statistical significance in predicting dual positivity. Based on these insights, we posited that while intratumoral inflammatory mediators (e.g., IL-6, TNF-α, and IL-1β) could modulate antitumor immunity and angiogenesis, thus influencing tumor progression and MVI onset [32], the overall patient systemic status often confounds systemic inflammatory indices in peripheral blood, limiting the clinical utility for predicting the MVI/Ki-67 status in HCC.

The expression of the OATP1B3 transporter on hepatocyte membranes could determine the uptake of Gd-EOB-DTPA in the HBP. Furthermore, reduced or absent contrast uptake could result in relative hypointensity on HBP imaging during hepatocellular carcinogenesis [33]. Additionally, gadoxetic acid-enhanced MRI nomograms effectively predicted HCC Ki-67 expression, with Ki-67 overexpression and T1 relaxation times (T1rt-Pre, T1rt-20 min) correlating significantly positively [34]. Meng et al. [35] also reported a high predictive value (AUC = 0.945) for multimodal MRI radiomics clinical features in preoperative MVI assessment, highlighting the diagnostic and prognostic potential of integrated imaging-clinical models. Moreover, You et al. [36] identified HBP T1WI-derived DL features as the primary predictors of MVI, while other meta-analyses confirmed the superior predictive performance of CT portal venous and MRI HBP sequences in MVI detection [37]. Herein, we used enhanced MRI HBP images for DL and radiomics feature extraction, offering two key advantages. First, HBP integrates hepatobiliary-specific information in a single phase, eliminating heterogeneity challenges associated with multiphasic data fusion and significantly reducing registration complexity and labor costs. Second, due to impaired gadoxetic acid uptake, most HCCs often exhibit marked hypointensity, enhancing tumor-liver contrast by 3–5-fold relative to conventional agents, thus enabling the precise delineation of small lesions (<1 cm). However, the radiomics model (RadScore) exhibited a noticeable performance decline from the training set (AUC: 0.787) to the external validation set (AUC: 0.708), reflecting a recognized drawback of hand-crafted radiomic features—their propensity for overfitting in limited cohorts and sensitivity to cross-institutional technical variations, suggesting possible capture of non-biological, site-specific noise. In contrast, the deep learning model (DL_RadScore) demonstrated greater robustness, with a more moderate drop from an AUC of 0.905 to 0.854, underscoring its ability to learn more generalizable representations and establishing it as the primary predictive component within the integrated model. The combination of these DL-derived features with clinical and radiomic parameters ultimately yielded a highly effective predictor for dual positivity, achieving an AUC of 0.939.Moreover, our findings aligned with previous research [38–41], providing a scholarly basis for preoperative assessment and postoperative adjuvant therapy selection, where high-risk patients (predicted dual positivity) may warrant aggressive interventions and shortened surveillance intervals. Notwithstanding the performance of our model, we acknowledge that multiphasic MRI confers additional hemodynamic information for HCC. Future research should therefore explore the integration of these features to determine whether they provide incremental predictive power, potentially bolstering the model’s comprehensiveness and clinical applicability. Furthermore, our study focused on predicting MVI and Ki-67 dual positivity as a binary outcome. This approach was clinically motivated by longitudinal evidence indicating that dual-positive patients constitute a distinct subgroup with a significantly elevated risk of early recurrence compared to those with single positivity or neither feature. However, it is important to note that the ‘non-dual-positive’ group is heterogeneous, encompassing patients with MVI-only, Ki-67-high only, or neither abnormality. While our model effectively identifies the highest-risk dual-positive patients, the prognostic implications for these intermediate subgroups may differ. Due to limited sample size in these specific subcategories within our cohort, a robust analysis of the model’s performance across all subgroups was not feasible. Future studies with larger, prospectively enrolled cohorts are warranted to further refine risk stratification and validate the model’s applicability across the entire spectrum of HCC pathological profiles.

Despite its valuable insights, this study had several notable limitations. First, the retrospective and dual-center design may introduce selection bias and limit generalizability, despite external validation; future large-scale, prospective, multi-center studies involving diverse populations are required. Second, by excluding patients with macrovascular invasion or extrahepatic metastases, our cohort primarily comprised early-stage HCC patients, which may not fully represent advanced disease subtypes; future validation in broader clinical populations is needed. Finally, although differences in scanners and protocols exist between centers, feature harmonization such as ComBat was not undertaken. However, a sensitivity analysis adjusting for site confirmed the robustness of our combined model. We intend to prioritize full harmonization in subsequent multi-center validation.

## Conclusion

We developed a nomogram model integrating MRI-based 2.5D DL, as well as radiomics and clinical features, with high predictive performance for MVI/Ki-67 dual positivity in HCC. Furthermore, we employed a single-sequence MRI framework, ensuring superior model stability, reliability, and generalizability, thus facilitating clinical implementation. Additionally, our multimodal approach leveraged complementary information sources, addressing various limitations of single-factor models, thereby yielding more robust and clinically actionable predictions. Moreover, the model could be employed in personalized risk stratification, enabling tailored treatment planning and prognostic evaluation. Meanwhile, waterfall plots were generated to visually demonstrate feature contributions, enhancing model interpretability. Overall, besides optimizing the model’s generalizability, our strategy could also enhance its applicability across diverse clinical settings.

## Supporting information

S1 File TRIPODAI_checklist. (PDF)

S2 File Data. (ZIP)

S3 File Supplementary Material S1 Raw Analysis Outputs. (ZIP)
